# Active monitoring versus an abduction device for treatment of infants with centered dysplastic hips: study protocol for a randomized controlled trial (TReatment with Active Monitoring (TRAM)-Trial)

**DOI:** 10.1186/s12887-023-04012-2

**Published:** 2023-04-29

**Authors:** Frederike E. C. M. Mulder, M. Adhiambo Witlox, Carmen D. Dirksen, Pieter Bas de Witte, Suzanne de Vos-Jakobs, Arno M. ten Ham, Melinda M. E. H. Witbreuk, Ralph Sakkers, Magritha (Margret) M. H. P. Foreman-van Drongelen, Simon G. F. Robben, TRAM-Trial Consortium, Christiaan J. A. van Bergen, Arnold T. Besselaar, Marieke Boot, Bart J. Burger, Florens Q. M. P. van Douveren, J. H. (Harjanneke) van Gelder, Yvon M.den Hartog, Iris Koenraadt-van Oost, Joost H. van Linge, Patrick G. M. Maathuis, Sophie Moerman, Renske M. Pereboom, Heleen M. Staal, M. C. (Marieke) van der Steen, Jaap J. Tolk, Diederik A. Vergroesen, A. (Elgun) V. C. M. Zeegers, Nina M. C. Mathijssen

**Affiliations:** 1 Department of Orthopedic Surgery, Care and Public Health Research Institute (CAPHRI), Maastricht University, Maastricht, the Netherlands; 2 Department of Orthopedic Surgery, Care and Public Health Research Institute (CAPHRI), Maastricht University Medical Center+, Maastricht, the Netherlands; 3 Department of Clinical Epidemiology and Medical Technology Assessment, Care and Public Health Research Institute (CAPHRI), Maastricht University Medical Center, Maastricht, the Netherlands; 4 Department of Orthopedics, Leiden University Medical Center, Leiden, the Netherlands; 5 Department of Orthopedics and Sports Medicine, Erasmus Medical Center - Sophia Children’s Hospital, Rotterdam, the Netherlands; 6 Department of Orthopedics, Sint Maartenskliniek, Nijmegen, the Netherlands; 7 Department of Orthopedic Surgery, OLVG, AUMC, Amsterdam, the Netherlands; 8 Department of Orthopedic Surgery, Wilhelmina Children’s Hospital, University Medical Center, Utrecht, the Netherlands; 9 Department of Hip Sonography, Diagnostiek Voor U, Eindhoven, the Netherlands; 10 Department of Radiology and Nuclear Medicine, Maastricht University Medical Center, Maastricht, the Netherlands; 11 Reinier Haga Orthopedic Center, Zoetermeer, the Netherlands; 12 Department of Orthopedics, Reinier de Graaf Hospital, Delft, the Netherlands

**Keywords:** Developmental dysplasia of the hip, DDH, Active monitoring, Abduction treatment

## Abstract

**Background:**

Developmental Dysplasia of the Hip (DDH) is one of the most common pediatric orthopedic disorders, affecting 1–3% of all newborns. The optimal treatment of centered DDH is currently under debate. This randomized controlled trial aims to study the (cost-)effectiveness of active monitoring versus abduction treatment for infants with centered DDH.

**Methods:**

This is a multicenter, parallel-group, open-label, non-inferiority randomized controlled trial studying the (cost-)effectiveness of active monitoring versus abduction treatment for infants with centered DDH in fourteen hospitals in the Netherlands. In total, 800 infants with centered DDH (Graf IIa-/IIb/IIc), aged 10–16 weeks, will be randomly allocated to the active monitoring or abduction treatment group. Infants will be followed up until the age of 24 months. The primary outcome is the rate of normal hips, defined as an acetabular index lower than 25 degrees on an antero-posterior radiograph, at the age of 12 months. Secondary outcomes are the rate of normal hips at the age of 24 months, complications, time to hip normalization, the relation between baseline patient characteristics and the rate of normal hips, compliance, costs, cost-effectiveness, budget impact, health-related quality of life (HRQoL) of the infant, HRQoL of the parents/caregivers, and parent/caregiver satisfaction with the treatment protocol.

**Discussion:**

The outcomes of this randomized controlled trial will contribute to improving current care-as-usual for infants with centered DDH.

**Trial registration:**

Dutch Trial Register, NL9714, registered September 6, 2021. https://clinicaltrialregister.nl/en/trial/29596

## Background

Developmental Dysplasia of the Hip (DDH) is one of the most common pediatric orthopedic disorders, affecting 1–3% of all newborns [1]. DDH encompasses a wide spectrum of growth disorders of neonatal and infant hips, ranging from mild acetabular dysplasia with a well-centered femoral head to severe acetabular dysplasia with dislocation of the femoral head [1, 2]. Untreated DDH can lead to chronic pain, gait abnormalities, and early-onset hip osteoarthritis [1–3]. It is estimated that up to 26% of hip arthroplasties in patients aged 40 years or younger are performed due to untreated or insufficiently treated hip dysplasia [4].

Centered DDH refers to centered hips with acetabular dysplasia, classified as Graf type IIa-/IIb/IIc with ultrasound evaluation (Table 1) [3, 5]. The optimal treatment of centered DDH is currently under debate. Abduction treatment is the most used treatment method for children under six months of age with centered DDH worldwide [6]. However, it is approximated that 85% of immature dysplastic hips will normalize at the age of three months without treatment [7]. Also, it is estimated that more than 80% of centered DDH hips (Graf IIa- to IIc) under six months will normalize without treatment [8]. This suggests that ultrasonography screening at a young age might introduce DDH overdiagnosis and possibly unnecessary treatment of immature hips that are not truly pathological.

**Table 1:** Ultrasonography classification of hip dysplasia by Graf [9]

Type	Alpha angle	Beta angle	Description
Ia	≥ 60	≤ 55	Normal, mature hip
Ib	≥ 60	> 55	Normal, mature hip
IIa +	50–59	> 55	Physiological immaturity, age appropriate (< 3 months old)
**IIa-**	**50–59**	** > 55**	**Maturational deficit (< 3 months old)**
**IIb**	**50–59**	** > 55**	**Delayed ossification (> 3 months old)**
**IIc**	**43–49**	** < 77**	**Critical zone**
D	43–49	> 77	Decentered
III	< 43		Decentered; perichondrium upward
IV	< 43		Decentered; perichondrium horizontal or downward

A few studies have compared active monitoring and abduction treatment for infants with centered DDH [10–15]. A recent review summarizes that there is no difference in acetabular index between active monitoring and abduction treatment for infants up to four months of age with centered DDH after three months [16]. However, the included six studies showed considerable methodological heterogeneity and two non-randomized studies were rated as serious risk of bias. A large randomized clinical trial, embedded in clinical practice, is warranted to validate the outcomes of the previous studies, and study the cost-effectiveness of active monitoring versus abduction treatment for infants with centered DDH. Therefore, the current randomized controlled trial aims to study the (cost)-effectiveness of active monitoring versus abduction treatment for infants with centered DDH.

## Methods/design

### Trial design and study setting

This is a multicenter, parallel-group, open-label, non-inferiority randomized controlled trial studying the (cost-)effectiveness of active monitoring versus abduction treatment for infants with centered DDH in fourteen hospitals in the Netherlands (Amphia Hospital, Amsterdam University Medical Center, Erasmus Medical Center, HagaZiekenhuis, Leiden University Medical Center, Maastricht University Medical Center, Máxima Medical Center, Medisch Spectrum Twente, Noordwest Ziekenhuisgroep, Onze Lieve Vrouwe Gasthuis, Reinier Haga Orthopedic Center, Sint Maartenskliniek, Spaarne Gasthuis, and University Medical Center Groningen). As part of the Dutch national DDH screening program, youth healthcare professionals will refer at-risk infants for an ultrasound evaluation at the age of three months (or sooner in case of suspected dislocation) [17]. Infants will be identified and recruited after diagnosis at outpatient clinics of the participating orthopedic departments. After diagnosis and assessment of the eligibility criteria, parents/caregivers will receive the written patient information leaflet. Parents/caregivers will return for a standard-care consultation to sign the informed consent after a reflection period of seven days. After written informed consent has been obtained, randomization and baseline measurements will be performed. Infants will be followed up until the age of 24 months.

### Eligibility criteria

The TReatment with Active Monitoring (TRAM)-Trial consists of infants aged 10–16 weeks who are diagnosed with centered DDH (Graf IIa-/IIb/IIc) with ultrasound evaluation. In case of bilateral DDH, only the hip with the most severe Graf classification at baseline will be included in the analyses. Additional inclusion criteria are good comprehension of the Dutch language and the written informed consent of the parents/caregivers. Exclusion criteria are hip instability (Graf type D/III/IV DDH), age < 10 weeks or > 16 weeks, (suspicion of) syndromic disease (e.g. arthrogryposis, cerebral palsy, Down syndrome), and prematurity (defined as a gestational age < 37 weeks).

### Interventions

**Fig. 1:**
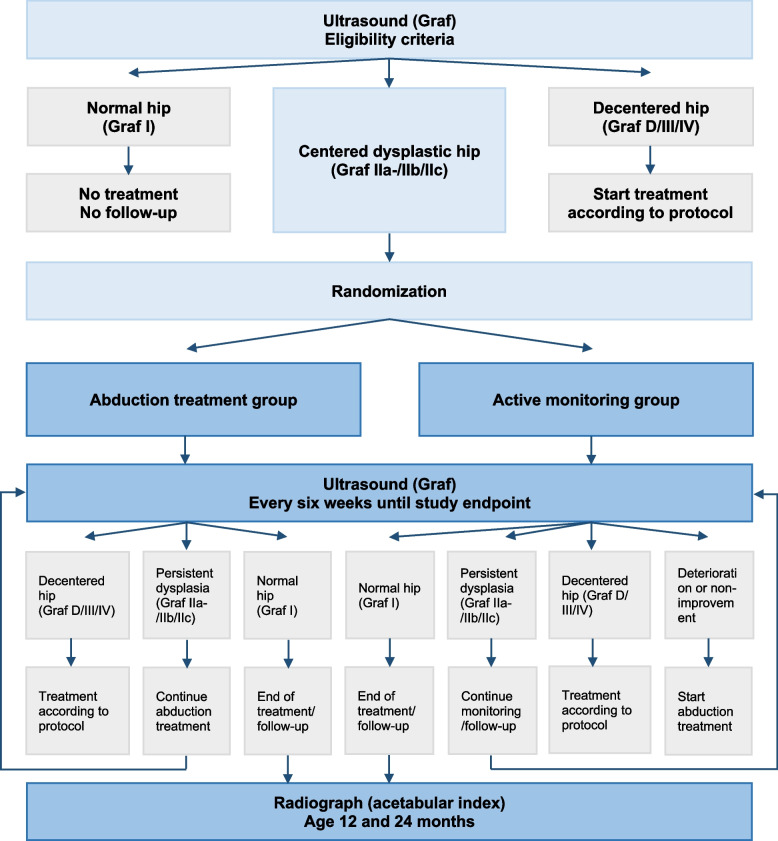
TRAM-Trial flowchart

In the active monitoring group (± delayed treatment), infants will not wear an abduction device and will be evaluated every six weeks with ultrasound and physical examination until reaching one of the endpoints (Table 2, Fig. 1). In the abduction treatment group, infants will wear an abduction device (e.g. Pavlik harness) and will be evaluated every six weeks with ultrasound and physical examination until reaching one of the endpoints (Table 2, Fig. 1). The Pavlik harness will be applied in 90–100 degrees of flexion of both hips and maximal comfortable abduction. Standard check-up after 1 and/or 2 weeks is advised after the start of Pavlik treatment. All infants in both groups will be evaluated at the age of 12 and 24 months.

**Table 2:** TRAM-Trial endpoints

Intervention	Endpoints	Treatment
Active monitoring	1. Hip normalization	Treatment will be discontinued as maximal results have been accomplished
	2. A total period of 18 weeks	Patients will receive treatment according to the standardized Dutch national protocol for usual care
	3. No improvement is observed on two consecutive imaging evaluations	
	4. Deterioration of the hip is observed with clinical examination or imaging. Deterioration for Graf type IIc is defined as worsening or not improving into Graf type IIb within 12 weeks	
	5. Inability to perform a reliable ultrasound evaluation because of progressive development of the ossific nucleus of the femoral head	Follow-up will be continued by obtaining radiographs
Abduction treatment	1. Hip normalization	Treatment with the dynamic abduction device will be discontinued as maximal results are accomplished
	2. No improvement is observed on two consecutive imaging evaluations	Patients will receive treatment according to the standardized Dutch national protocol for usual care
	3. Deterioration of the hip is observed with clinical examination or imaging	
	4. The infant is too strong for the dynamic abduction device	Abduction treatment will be continued using a static abduction device (e.g. CAMP device) until 1, 2 or 3 is accomplished
	5. Inability to perform a reliable ultrasound evaluation because of progressive development of the ossific nucleus of the femoral head	Follow-up will be continued by obtaining radiographs

During baseline and follow-up evaluations, physical examination consists of weight measurements, asymmetric skin fold evaluation, knee height (Galeazzi test), degrees of hip abduction in flexion, and Barlow and Ortolani tests. At the age of 12 and 24 months, physical examination consists of degrees of hip abduction and leg length evaluation. Ultrasounds will be performed every 6 weeks according to the Graf method until hip normalization or technically restricted due to the appearance of the ossific nucleus of the femoral head. For the latter case, radiographs will be obtained in further follow-up. At the age of 12 and 24 months, pelvic radiographs will be performed according to a standardized protocol. Ultrasounds and radiographs will be assessed by local pediatric radiologists at the participating centers.

### Outcomes

#### Primary outcome

The primary outcome is the rate of normal hips, defined as an acetabular index lower than 25 degrees on an antero-posterior radiograph, at the age of 12 months.

#### Secondary outcomes

The rate of normal hips, defined as acetabular index lower than 25 degrees on an antero-posterior radiograph, at the age of 24 months will be studied. Also, the relation between baseline patient characteristics and the rate of normal hips will be explored. Included patient characteristics are gender, birthweight, initial alpha angle, initial beta angle, laterality (right or left, unilateral or bilateral), history of DDH in relatives, breech presentation, swaddling, twin birth, parents/caregivers’ education level, ethnicity, range of abduction in flexion (degrees), and whether the child is the firstborn child of the parents/caregivers. Accordingly, the time to hip normalization is studied. Complications during the follow-up period will be examined. The compliance of wearing the abduction device in the abduction treatment group will be assessed using visual inspection of the abduction device and a parent/caregiver-question on the number of hours that the abduction device has been worn in the last 24 hours. Furthermore, resource use and costs will be assessed using standardized cost-questionnaires. The health-related quality of life (HRQoL) of the infant will be measured with the Visual Analogue Scale (EQ-VAS) of the youth version of the EQ-5D (EQ-5D-Y) and the Infant and Toddler Quality of Life Questionnaire Short Form (ITQOL-SF47), filled out by one parent/caregiver. Last, the HRQoL of one parent/caregiver will be measured with the EQ-5D-5L and parent/caregiver satisfaction with the treatment process will be assessed with the Visual Analogue Scale.

### Participant timeline

A participant timeline of measurement moments of the TRAM-Trial is shown in Table 3.

**Table 3:**
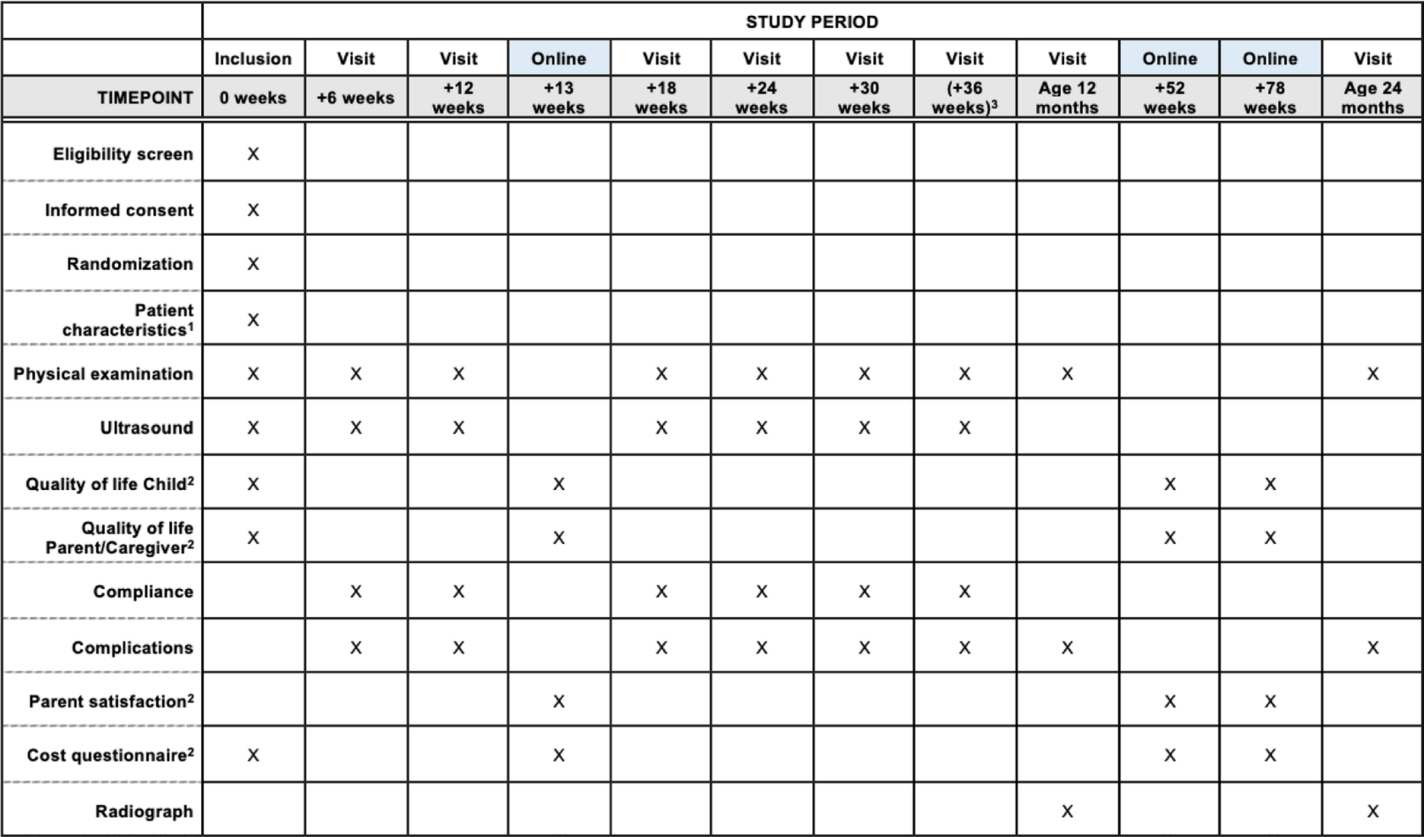
Study procedures and questionnaires at follow-up time points of the TRAM-Trial

^1^ Patient characteristics: gender, birthweight, laterality (right/left, unilateral/bilateral), history of DDH in relatives, breech presentation, swaddling, twin birth, socio-economic status parents/caregivers, ethnicity, firstborn, age of parents/caregivers.

^2^ Extra compared to care-as-usual.

^3^ + 36 weeks measurements are only performed if the infant was 10 weeks of age at inclusion.

### Sample size and recruitment

We hypothesize that the rate of normal hips of infants treated with active monitoring is not lower than the rate of normal hips of infants treated with an abduction device. Based on the study results of Ömeroglu et al. [18], we assume a rate of normal hips of 80%. The inferiority margin was set to 10%, based on consensus in the study group (70% adequate treatment in the active monitoring group, 80% adequate treatment in the abduction treatment group). With an alpha of 0.05 and power of 90%, the sample size is 370 infants per group. Accounting for 10% lost to follow up, a total of 800 infants (400 per group) is warranted.

### Allocation and blinding

Infants will be randomly allocated to the active monitoring or abduction treatment group. Randomization will be performed by a computer-generated randomization schedule. Block randomization will be used, stratifying for Graf type IIa-/IIb/IIc and participating center. Due to the nature of this study, parents/caregivers and treating physicians cannot be blinded. However, the researchers performing the data analyses will be blinded.

### Data collection methods

Data collection is embedded in standard-care follow-up moments according to the Dutch DDH guideline (Table 3) [17]. At the enrollment consultation, baseline patient characteristics, physical examination data, and ultrasonographic measurements will be collected by the physician or researcher. Infants and parents/caregivers will return for a consultation at the orthopedic department every six weeks (± 1 week) to collect physical examination data, ultrasonographic measurements, and data on compliance and complications, until one of the endpoints (Table 2).

Additionally, baseline cost- and HRQoL questionnaires will be sent to the parents/caregivers via email. One parent/caregiver is asked to digitally fill in the cost- and HRQoL questionnaires at baseline and after 3, 12, and 18 months. The parent/caregiver will fill in two HRQoL questionnaires, one for the infant and one for the parent/caregiver. If the parent/caregiver has not filled in the questionnaires, a digital reminder will be sent after two weeks, and the parent/caregiver will be contacted by phone after four weeks.

At the age of 12 and 24 months (± 1 month), the infants and parents/caregivers will return for a consultation at the orthopedic department to collect physical examination data, radiograph measurements, and data on complications.

All members of the TRAM-Trial team will be protocol trained for standardized evaluation of included subjects.

### Data management

A data management plan was constructed. All data will be pseudonymized prior to entry into the Castor database (Castor, Amsterdam, the Netherlands). The local principal investigators and research nurses in the participating centers have access to the local code key. Two PhD-students have access to the code key of all participating centers. Source documents will be stored locally at the participating centers. All ultrasounds and radiographs will be pseudonymized and stored at MUMC + . The local principal investigators and research nurses in the centers will have access to local source documents. The two PhD-students, monitors, and the Health and Youth Care Inspectorate will have access to all source documents. All data will be stored for 15 years.

### Statistical methods

Statistical analyses will be performed according to the intention-to-treat principle and per-protocol approach. The difference in rate of normal hips between active monitoring and abduction treatment at the age of 12 and 24 months will be analyzed with chi-square tests. The non-inferiority margin will be set to 10% and non-inferiority will be demonstrated if one side of the 95% confidence interval lies outside the non-inferiority margin.

A Kaplan Meyer analysis will be performed to study the time to hip normalization, with events defined as Graf I or acetabular index lower than 25 degrees at 12 months or acetabular index lower than 25 degrees at 24 months. Differences in survival curves will be studied and tested for statistical significance with the Log Rank test. The relation between patient characteristics and the rate of normal hips at the age of 12 and 24 months will be studied with prediction models. Using backward elimination, a multivariable cox regression model will be constructed. The Receiver Operating Characteristic (ROC) curve and Area Under the Curve (AUC) will be used as performance parameters, goodness-of-fit will be determined by inspection and publication of the calibration plot and publication of discrimination parameters, and internal validation of the model will be performed with bootstrapping. Compliance will be presented using descriptive analysis. The difference in the number of complications will be studied with a t-test (total number of complications) and chi-square test (number of infants with a complication). Differences in quality of life and parent/caregiver satisfaction will be determined with generalized estimated equations (GEE). The statistical analyses will be performed with SPSS version 25 (IBM SPSS Statistics for Windows; Armonk, New York: IBM Corp.).

#### Cost effectiveness analysis

A trial-based cost-effectiveness analysis will be performed from a societal- and healthcare perspective with a time horizon of 24 months and according to the Dutch Manual for Cost Analysis in Health Care Research [19]. Incremental cost-effectiveness ratios will be calculated as societal cost per infant QALY (societal perspective) and healthcare cost per additional infant with a normal hip (healthcare perspective). Cost-effectiveness acceptability curves will visualize the cost-effectiveness probability for a range of threshold values. Total costs will be calculated by multiplying all resource use related to hip dysplasia with the costs per unit. Resource use will be extracted from hospital electronic patient files and standardized cost-questionnaires with a recall period of three months. If available, standardized, national cost-prices will be used [19]. If not available, hospital-specific- or published unit-prices will be used. The friction cost method will be used for productivity losses by parents/caregivers. The healthcare-perspective analysis will be based on the proportion of infants with a normal hip at the age of 24 months. The societal-perspective analysis will be based on the EQ-VAS of the EQ-5D-Y, which will be administered at baseline and after 3, 12, and 18 months, and will be filled out by one parent/caregiver. The base-case cost-utility analysis is based on the societal cost per QALY of the infant, obtained from the VAS of the EQ-5D-Y. Additionally, a secondary cost-utility analysis will calculate the cost per QALY of the parent/caregiver, obtained from the EQ-5D-5L. Costs and effects occurring twelve months after study inclusion will be discounted at 4% and 1.5% respectively according to the Dutch manual [19]. Uncertainty will be addressed with standard bootstrap- and sensitivity analyses.

#### Budget impact analysis (BIA)

A BIA will be performed in accordance with the Dutch manual and the ISPOR guidelines [19, 20]. The BIA addresses the financial consequences related to the implementation of active monitoring and thus its affordability. A simple decision analytical model will be built. Input parameters will be based on study results, national prevalence data, unit prices, and tariffs obtained in the cost-effectiveness analysis and available literature. The BIA will be performed from different perspectives (e.g. health care budgetary, health insurance) with a five-year time horizon. The BIA target population will be similar to the study population. Optimistic and pessimistic scenarios will be compared to investigate various levels of implementation (e.g. 100%, 50%) of active monitoring in the Netherlands, as well as the swiftness of implementation (e.g. within 1 years, 2 years). No discounting will be applied.

### Monitoring

Data monitoring in all participating centers will be performed according to the data monitoring plan by the Clinical Trial Center Maastricht (CTCM). CTCM will perform the data monitoring independently from the Sponsor and there are no competing interests. All adverse events related to the trial procedure will be recorded. All serious adverse events (SAEs) will be reported to the Sponsor and to the medical ethics committee via the national web portal ToetsingOnline. SAEs that are life threatening or result in death will be reported within seven days of first knowledge, with a maximum of eight days to complete the initial report. Other SAEs will be reported within fifteen days after first knowledge.

### Ethics and dissemination

The TRAM-Trial was approved by the Medical Ethics Committee of Maastricht University Medical Center + (MUMC +) and Maastricht University, the Netherlands (METC 21–036). Important protocol modifications will be communicated to all relevant parties. Written informed consent of the infant will be obtained from both parents/caregivers. Additionally, informed consent of one parent/caregiver will be obtained from the parent/caregiver who will fill in the questionnaires. All data will be pseudonymized prior to storage in the Castor database. All participating hospitals jointly own the TRAM-Trial data. The use of the TRAM-Trial data outside the scope of the study protocol has been described in a Collaboration Agreement. Trial results will be reported in manuscripts that will be handed in for publication to peer-reviewed journals. All results will be communicated to relevant parties.

## Discussion

DDH is one of the most common pediatric orthopedic disorders, with potential unfavorable outcomes, including chronic pain, gait abnormalities, and early-onset hip osteoarthritis when left untreated [1–3]. Currently, abduction treatment is the most used treatment method for children under six months of age with centered DDH [6]. However, it has been suggested that centered DDH hips tend to normalize without treatment during growth [7, 8]. Therefore, ultrasonography at a young age might introduce overtreatment of immature centered DDH hips that are not truly pathological. This randomized controlled trial will assess whether active monitoring of infants with centered DDH (Graf type IIa-/IIb/IIc) does not result in a lower proportion of infants with normal hips at the age of 12 months compared to abduction treatment (a non-inferiority study). We hypothesize that active monitoring is not inferior to abduction treatment for infants with centered DDH aged 10–16 weeks with regards to the rate of normal hips, and that it is cost-effective. The outcomes of the TRAM-Trial will contribute to improving current care-as-usual for infants with centered DDH.

## Data Availability

The datasets used and/or analyzed during the current study are available from the corresponding author on reasonable request.

## Abbreviations

TRAM: TReatment with Active Monitoring
DDH: Developmental dysplasia of the hip
HRQoL: Health-related quality of life
AVN: Avascular necrosis
EQ-VAS: EuroQol visual analogue scale
EQ-5D-Y: EuroQol five dimensions health questionnaire youth
GEE: Generalized estimated equations
ROC: Receiver operating characteristic
AUC: Area under the curve
